# A retrospective cohort investigation of seroprevalence of Marburg virus and ebolaviruses in two different ecological zones in Uganda

**DOI:** 10.1186/s12879-020-05187-0

**Published:** 2020-07-01

**Authors:** Luke Nyakarahuka, Ilana J. Schafer, Stephen Balinandi, Sophia Mulei, Alex Tumusiime, Jackson Kyondo, Barbara Knust, Julius Lutwama, Pierre Rollin, Stuart Nichol, Trevor Shoemaker

**Affiliations:** 1 Arbovirology, Emerging and Re-emerging Diseases, Uganda Virus Research Institute , Entebbe, Uganda; 2 Department of Biosecurity, Ecosystems and Veterinary Public Health, College of Veterinary Medicine, Animal Resources and Biosecurity, Makerere University, Kampala, Uganda; 3 Centres for Disease Control and Prevention, Atlanta, USA

**Keywords:** Marburg virus disease, Ebola virus disease, Filovirus, Seroprevalence, Epidemiology, Ebolaviruses, Uganda, ELISA

## Abstract

**Background:**

Uganda has experienced seven Ebola Virus Disease (EVD) outbreaks and four Marburg Virus Disease (MVD) outbreaks between 2000 and 2019. We investigated the seroprevalence and risk factors for Marburg virus and ebolaviruses in gold mining communities around Kitaka gold mine in Western Uganda and compared them to non-mining communities in Central Uganda.

**Methods:**

A questionnaire was administered and human blood samples were collected from three exposure groups in Western Uganda (gold miners, household members of miners, non-miners living within 50 km of Kitaka mine). The unexposed controls group sampled was community members in Central Uganda far away from any gold mining activity which we considered as low-risk for filovirus infection. ELISA serology was used to analyse samples, detecting IgG antibodies against Marburg virus and ebolaviruses (filoviruses). Data were analysed in STATA software using risk ratios and odds ratios.

**Results:**

Miners in western Uganda were 5.4 times more likely to be filovirus seropositive compared to the control group in central Uganda (RR = 5.4; 95% CI 1.5–19.7) whereas people living in high-risk areas in Ibanda and Kamwenge districts were 3.6 more likely to be seropositive compared to control group in Luweeero district (RR = 3.6; 95% CI 1.1–12.2). Among all participants, filovirus seropositivity was 2.6% (19/724) of which 2.3% (17/724) were reactive to Sudan virus only and 0.1% (1/724) to Marburg virus. One individual seropositive for Sudan virus also had IgG antibodies reactive to Bundibugyo virus. The risk factors for filovirus seropositivity identified included mining (AOR = 3.4; 95% CI 1.3–8.5), male sex (AOR = 3.1; 95% CI 1.01–9.5), going inside mines (AOR = 3.1; 95% CI 1.2–8.2), cleaning corpses (AOR = 3.1; 95% CI 1.04–9.1) and contact with suspect filovirus cases (AOR = 3.9, 95% CI 1.04–14.5).

**Conclusions:**

These findings indicate that filovirus outbreaks may go undetected in Uganda and people involved in artisan gold mining are more likely to be exposed to infection with either Marburg virus or ebolaviruses, likely due to increased risk of exposure to bats. This calls for active surveillance in known high-risk areas for early detection and response to prevent filovirus epidemics.

## Background

**Fig. 1:**
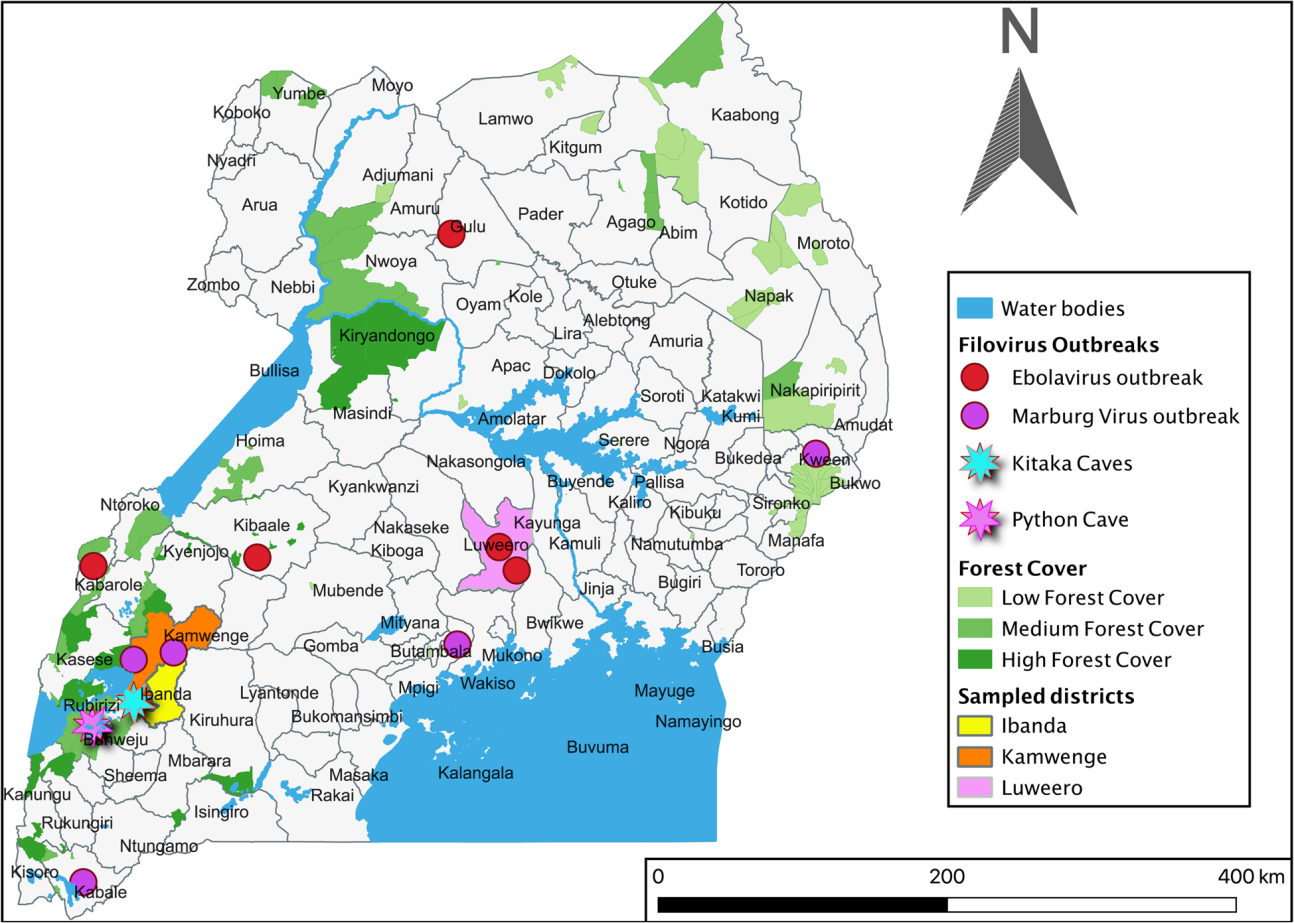
Reported filovirus outbreaks, cohort investigation districts, water and forest cover of Uganda

Viruses in the genera *Ebolavirus* and *Marburgvirus* belong to the family *Filoviridae* and cause classical viral haemorrhagic fevers (VHFs) in humans, which are associated with high morbidity and mortality and pose a serious threat to human and animal populations in endemic countries. Uganda reported 11 filovirus outbreaks from 2000 to 2019. These include seven EVD outbreaks caused by Sudan virus (6 outbreaks) and Bundibugyo virus (one outbreak) and four Marburg virus disease (MVD) outbreaks caused by Marburg virus and Ravn virus [1]. In Ibanda and neighbouring Kamwenge districts of western Uganda, there were two documented outbreaks of MVD [2, 3] including one in which cases were gold miners in Kitaka cave [3]. Previous studies of bats sampled from Kitaka and python caves have shown *Rousettus aegyptiacus* bats to be the known reservoir for Marburg virus [4–6]. In 2012, the MVD outbreak investigations in Ibanda district traced the outbreak’s origin to villages near Kitaka mines where artisanal gold mining is practised [2]. We designed an investigation to better understand the possible link between artisanal gold mining activities in Kitaka and the transmission of Marburg virus and ebolaviruses in Ibanda and Kamwenge districts. We compared these communities with those in Luweero district where there are no mining operations or bat-inhabited caves, no previously identified human cases of MVD, and there is a different ecological zone. Although there have been outbreaks of EVD in Luweero in 2010 and 2012, investigations did not reveal any potential sources of spillovers in the Luweero area in terms of bat-inhabited caves and forested areas (Fig. 1) and we hypothesise that these cases could have been imported from other hotspots in Uganda.

## Methods

### Sampling sites, population, and hypothesis

Participants were sampled from Ibanda, Kamwenge and Luweero districts (Fig. 1). The bat-inhabited Kitaka mines are located within the boundary of Ibanda and Kamwenge within Kasyoha-Kitomi Forest Reserve. The caves were created as a resultant of abandoned decommissioned gold mines during colonial times, however, some minimal artisanal mining still happens update. Workers in the mines and communities that live in and around this reserve were considered to be at higher risk of exposure to filovirus infection because of the *Rousettus aegyptiacus* bats that live in the mines, a known reservoir for Marburg virus. A comparison group in Luweero district was chosen as a control, unexposed group because it is in the Central region of the country far from Kitaka and any other mines, and we hypothesized that *Rousettus aegyptiacus* bats may not inhabit this region due to lack of suitable habitat, no previously reported MVD cases, and therefore inhabitants would mostly likely be at low risk for exposure to filoviruses. Despite EVD being reported in Luweero district in 2011 and 2012, the ecology of this place is different from that of Ibanda and Kamwenge districts as it is not forested and not have suitable habitat for the putative filovirus reservoirs (Fig. 1). The Luweero population was used as an unexposed group as the investigation of the previous cases of EVD did not reveal any possible sources of virus spillover from wildlife in Luweero.

### Sampling procedure and inclusion criteria

We sampled three groups of exposed individuals in Ibanda and Kamwenge districts that included: 1) Artisanal miners and persons that worked in the Kitaka mines from 2007 to 2015, 2) Members of the household or family housing compound of a miner during the period that the miner was actively working in Kitaka mines, 3) Members of households that resided within a 50 km radius from any open mining site associated with Kitaka mines, and that were not included in above groups 1 or 2. This third group also acted as a control for groups 1 and 2 above to assess the risk for filovirus infection between people with direct or indirect exposure to the mine versus living near it. We then sampled the 4^th^ group of unexposed individuals who are residents of Luweero district. The sample size needed for this study was calculated to be 500 individuals in total. This calculation included 100 persons in group ‘1’ (as listed in «Inclusion Criteria» above), 200 persons between groups ‘2’ and ‘3’ and 200 persons in group ‘4’. This calculation was done estimating a 15% prevalence of Marburg virus exposure in group ‘1,’ 5% prevalence in groups ‘2’ and ‘3,’ and 0.5% prevalence of exposure in group ‘4,’ as well as a 95% confidence interval, 80% power, and a ratio of 2 controls to each exposed person. However, because of the high response rate (144.8%) during participant recruitment, a total of 724 individuals were sample (group 1 = 161, group 2 = 138, group 3 = 134 and group 4 = 291). For groups 1 and 2, a purposive sampling procedure was used with a snowball approach. Participants were questioned to determine those currently working or those who used to work in Kitaka mines. The discovered miners were further questioned to identify additional miners or ex-miners. All discovered miners and their family and household members who were willing to participate were included in the project. For groups 3 and 4, a two-stage cluster sampling design was used, with a random selection of five sub-counties in each sampling area (sub-counties within 50 km of Kitaka mines in Ibanda/Kamwenge for group 3, entire Luweero district for group 4), followed by the random selection of three villages within those sub-districts. In selected villages, the investigators travelled to the location of the main trading post at the village’s centre, and participants were chosen following the EPI method [7]. Sampling was done in January and February 2015 in all three districts of Kamwenge, Ibanda and Luweero. Participants that consented to inclusion in the study were interviewed to complete a risk factor questionnaire and provided their answers verbally. One blood sample (4 ml) was collected from each participant for serological testing for filovirus (marburgviruses, Sudan virus, Bundibugyo virus and Ebola virus) IgG by ELISA at Uganda Virus Research Institute (UVRI)/Centres for Disease Control (CDC) VHF laboratory, at Entebbe Uganda.

### Data management and laboratory analysis

Data were entered in Epi Info 7 and analysed using STATA (StataCorp. 2015. Stata Statistical Software: Release 14. College Station, TX: StataCorp LP). Risk ratios (RR) were calculated to evaluate the risk of filovirus seropositivity between different exposure groups and the unexposed control group, and to compare the risk for seropositivity between hypothesized higher-risk exposure groups (miners and their household members) to the lower risk exposure group (non-miners living within 50 km of the mines). To investigate significant risk factors for seropositivity among all participants (exposed and unexposed groups), we computed Odds Ratios (OR), which provide a reasonable estimation of the Risk Ratio (RR) since the exposure-outcome is less than 10% [8]. We controlled for potential confounding by adjusting for sex, age, and education level by computing the adjusted odds ratio (AOR).

All the samples collected were tested for the presence of IgG antibodies by Enzyme-Linked Immunosorbent Assay (ELISA), which was validated by US Centres for Disease Control and Prevention (CDC) on known positive and negative human samples with a sensitivity of more than 90% and specificity of more than 90% [9].

Briefly, a gamma-irradiated lysate of Vero cells (made in-house by US CDC Atlanta) infected with either Sudan virus, Bundibugyo virus, Ebola virus or Marburg virus was used as positive antigen whereas the negative or control antigen had uninfected Vero cells. 100 μl of positive antigen diluted in Phosphate Buffered saline (Sigma Life Science Inc., Missouri USA) (Marburg Ag 1:3000 and Ebola Ag 1:2000 Dilutions) was applied on the upper half of the solid phase of a polyvinyl chloride microtiter plate (Thermo Fisher Scientific, USA) and the lower half coated with 100 μl of negative/control antigen in PBS then incubated at 4 °C overnight. Unbound antigen was removed from the well by washing three times with PBS-Tween (Research Products International Corp, IL, USA). Samples were diluted 1:100 and 4-fold through 1:6400 in 5% skimmed milk in PBS-Tween and allowed to bind to the antigen. After washing, an anti-human IgG conjugated to horseradish peroxidase (HRPO) was added and allowed to bind. The plates were washed and the substrate ABTS (2.2′-Axinobis 3-ethylbenzothiazoline-6-sulfonic acid-diammonium salt-Seracare Life sciences Inc.MA, USA) was added which in the presence of HRPO (Seracare Life sciences Inc. MA, USA) and hydrogen peroxide, is converted from a colourless liquid to an intense green colour with a maximum light absorption at 410 nm. The amount of colour developed is proportional to the number of IgG antibodies which has bound to the antigen on the solid phase. OD values at 410 nm were recorded on a microplate spectrophotometer. The OD value of the control antigen-coated well was subtracted from its corresponding viral antigen-coated well to yield adjusted OD value. A sample was considered positive when the adjusted OD value of either the 1:400, 1:1600 or 1:6400 dilution was greater than 0.2 and the sum OD value was greater than 0.95. A panel of 1 or 2 negative control sera and 2 or 3 positive control sera were run each time the assay was used. Positive samples were retested against three circulating viruses (Sudan virus, Bundibugyo virus and Ebola virus) to assess cross-reactivity.

## Results

Overall, we sampled 724 individuals, 291 (40.2%) from the low-risk exposure region in Central Uganda (Luweero district) and 433 from the high-risk exposure region in Western Uganda (Ibanda and Kamwenge districts), including 161 miners (22.2%), 138 miner household members (19.1%), and 134 non-miners living within 50 km of the mines (18.5%) (Table 1). The mean age of all participants was 36.3 years (SD = 14.8, 95% CI = 35.2–37.4), the median age was 33.0 years (range 3–82), and 85.6% (620/724) of the sampled people were ≥ 20 years. Males represented 54.1% (391/724) of the people sampled. There were no significant differences in age, sex, and education level between the three exposure groups and the non-exposed group. Most participants were farmers (67.7%) whereas 71.6% of participants had primary school education or less.

**Table 1:** Summary of participant cohort groups and corresponding seroprevalences and risk ratios

Study Cohorts	Number sampled (*N* = 724)	Marburg virus seroprevalence (%)	Sudan virus (SUDV) seroprevalence (%)	Filovirus seroprevalence (%)	Filovirus (Marburg & SUDV) seroprevalence Risk Ratio (95%CI)
Low-Risk group (Luweero district)	291 (40.2%)	0	3 (1.1%)	3 (1.1%)	Reference^a^
High risk groups (Ibanda and Kamwenge districts)	433 (59.8%)	1 (0.2%)	15 (3.5%)	16 (3.7%)	3.6 (1.1–12.2) ^c^
Miners only	161 (22.2%)	1 (0.6%)	8 (4.9%)	9 (5.6%)	5.4 (1.5–19.7) ^c^
Family/household member of miner^d^	138 (19.1%)	0	4 (2.9%)	4 (2.9%)	2.8 (0.64–12.4)
Non-miners within 50 km of Kitaka mine ^b^	134 (18.5%)	0	3 (2.2%)	3 (2.2%)	2.2 (0.44–10.6)

^a^All other groups (exposure groups) were compared to the unexposed group as control

^b^Seropositivity among people who live within 50 km of Kitaka cave was not significantly different from miners or their family members

^c^Statistically significant

^d^One person seropositive for SUDV in this exposure group was also seropositive for Bundibugyo virus

In total, 2.6% (19/724) individuals tested had IgG antibodies against filoviruses. One person in the miner’s exposure group had IgG antibodies against Marburg virus (0.1%, 1/724). Seventeen individuals (17) were reactive against Sudan virus IgG antigens only (2.3%, 17/724), showing no cross-reactivity of the assay between Sudan Virus and Ebola virus. However, one person who was a family member of a miner had IgG antibodies to Sudan virus and Bundibugyo virus showing low cross-reactivity between the Sudan virus and Bundibugyo virus. No individuals had IgG antibodies against Ebola virus (EBOV).

The three combined high-risk exposure groups in Ibanda and Kamwenge district in Western Uganda had higher filovirus positivity at 3.7% (16/433) compared to low-risk exposure group in Central Uganda at 1.1% (3/291). Specifically, artisanal gold miners who enter bat-inhabited caves had a higher seroprevalence of 5.6% (9/161) compared to non-miners in central Uganda at 1% (3/291) and had 5.4 times the risk of being seropositive for filoviruses compared to the unexposed group in central Uganda (RR = 5.4, 95% CI 1.5–19.7). Miner family/household members and non-miners living within 50 km of the mines were not significantly more likely to be filovirus seropositive than the non-exposed participants in Luweero district (Table 1). Also, miners and miner family/household members were not more likely to be filovirus seropositive when compared to non-miners living within 50 km of the mines (Table 1).

Significant risk factors for filovirus seropositivity among all participants (combined exposure and unexposed groups), include male sex (AOR = 3.1; 95% CI 1.01–9.5), going inside mines (AOR = 3.1; 95% CI 1.2–8.2), cleaning corpses (AOR = 3.1; 95% CI 1.04–9.1) and contact with EVD/MVD suspects (AOR = 3.9; 95% CI 1.04–14.5) (Table 2). Frequent travels (once a month) outside a persons’ home district was shown to be protective (AOR = 0.3; 95% CI 0.1–0.7).

**Table 2:** Risk factors for filovirus seropositivity among all participants

Variable	Category	Filovirus IgG Seropositive (%)	Filovirus IgG Seronegative (%)	^**b**^Adjusted OR (95%CI)
**Total participants (*n*** **= 724)**		19 (2.6%)	705 (97.4%)	
**Age (years)**	< 20	1 (0.96%)	103 (99.04%)	
	> 20	18 (2.9%)	602 (85.4%)	1.9 (0.2–14.7)
**Gender**	Female	4 (1.2%)	329 (98.8%)	
	Male	15 (3.8%)	376 (96.2%)	3.1 (1.01–9.5) ^a^
**Education**	Never	5 (4.1%)	117 (95.9%)	
	Primary	10 (2.5%)	387 (97.5%)	0.4 (0.1–1.3)
	Secondary	4 (2.2%)	182 (97.9%)	0.3 (0.1–1.4)
	Tertiary	0 (0%)	19 (100%)	
**District**	Luweero	3 (1.1%)	288 (98.9%)	
	Ibanda	9 (3.7%)	235 (96.3%)	2.4 (0.6–10.2)
	Kamwenge	7 (3.7%)	182 (96.3%)	2.4 (0.6–10.7)
**Famer**	No	5 (2.2%)	222 (97.8%)	
	Yes	14 (2.9%)	461 (97.5%)	1.3 (0.4–3.6)
**Go inside mines**	No	10 (1.7%)	576 (98.3%)	
	Yes	9 (6.5%)	129 (93.5%)	3.1 (1.2–8.2) ^a^
**Contact with bats in mines**	No	4 (3.5%)	111 (96.5%)	
	Yes	5 (8.2%)	56 (91.8%)	1.9 (0.5–7.4)
**Own Domestic animals**	No	3 (1.8%)	159 (98.2%)	
	Yes	16 (2.8%)	546 (97.2%)	1.3 (0.4–4.8)
**Contact with Animals**	No	2 (1.2%)	161 (22.8%)	
	Yes	17 (3.1%)	544 (96.9%)	3.7 (0.4–36.3)
**Hunting**	No	14 (2.3%)	582 (97.7%)	
	Yes	5 (3.9%)	123 (96.1%)	1.1 (0.4–3.4)
**Contact with dead animals**	No	16 (2.5%)	621 (97.5%)	
	Yes	3 (4.5%)	63 (95.5%)	1.4 (0.4–4.9)
**Eat bush meat**	No	6 (1.6%)	371 (98.4%)	
	Yes	13 (3.7%)	334 (96.3%)	2.0 (0.7–5.6)
**Cleaning of the dead body**	No	12 (2%)	590 (98.0%)	
	Yes	5 (5.8%)	81 (94.2%)	3.1 (1.04–9.1) ^a^
**MVD reported in the village**	No	11 (1.9%)	563 (98.1)	
	Yes	8 (5.3%)	142 (94.7%)	2.2 (0.8–6.2)
**Contact with EVD/MVD suspects**	No	16 (2.3%)	677 (97.7%)	
	Yes	3 (9.7%)	28 (90.3%)	3.9 (1.04–14.5) ^a^
**Frequently travels**	No	7 (5.9%)	112 (94.1%)	
	Yes	12 (2%)	593 (98.0%)	0.3 (0.1–0.7) ^a^
**Go to the Forest frequently**	No	4 (1.6%)	240 (98.4%)	
	Yes	15 (3.1%)	465 (96.9%)	2.(0.7–6.1)
**Wash fruits before eating**	No	13 (3.0)	417 (96.9%)	
	Yes	6 (2.2%)	264 (97.8%)	0.9 (0.3–2.4)
**Reported of bats in the house**	No	4 (1.3%)	313 (98.7%)	
	Yes	15 (3.7%)	392 (96.3%)	2.5 (0.8–7.8)

^a^statistically significant

^b^Adjusted for gender, age and education level

## Discussion

Comparing the two groups of people, one living near an ecosystem of the bat inhabited caves and forest reserves in western Uganda (exposed) and another in savanna rangeland in central Uganda (unexposed), we see that the risk of being filovirus seropositive is higher specifically among miners by at least five times compared to the unexposed group, but not higher among household members of miners or people living near the mines. Therefore, filovirus exposure risk is specifically associated with exposures occurring in the mines themselves, likely exposure to bats or their excrement or body fluids. As has been reported before, the decommissioned bat-occupied Kitaka mines where the exposed population is centred is inhabited by bats of species *Rousettus aegyptiacus* that are the known reservoirs for Marburg virus [3, 5, 6].

We expected a higher seroprevalence against Marburg virus than ebolaviruses, but the opposite was observed with ebolaviruses seroprevalence being higher than Marburg virus. Whereas it has been confirmed during previous investigations that bats occupying the mines are actively infected with Marburg virus and had been associated with two MVD outbreaks [3, 10], no outbreak of EVD has been reported in this region. It was therefore surprising to find higher seroprevalence to ebolaviruses instead of the expected Marburg virus. We cannot clearly explain why Marburg virus seroprevalence is lower than that of ebolaviruses, but this is consistent with other studies where the two pathogens have been tested [11–17]. One of the explanations could be that the antibodies for Marburg virus are not as long-lasting compared to those of ebolaviruses. Studies in Egyptian rousette bats have shown that IgG antibodies against Marburg virus do not last more than 3 months [18]. In the same study, bats were protected by secondary immunity when reinfected with Marburg virus. If a similar mechanism occurs in humans is yet to be fully understood, and further studies of the immune responses in MVD survivors in Uganda are needed. Another possibility for this finding is there could be a reservoir for ebolaviruses or another closely related filovirus in Kitaka mines and/or inhabiting the area around the Kasyoho-Kitomi reserve ecosystems to which these individuals were exposed, especially the gold miners. This area is near Queen Elizabeth National Park, and so there is a possibility of having an unknown reservoir of ebolaviruses in the game reserve that has not been previously identified.

Another filovirus serological study by Nkoghe et al. (2011) in rural Cameroon and Gabonese populations where the prevalence of Ebola virus was higher in populations near forests [19]. A second study in Gabon found that pygmies, who are forest dwellers, had a higher percentage of ebolavirus seroprevalence than other populations at 7.02% compared to non-pygmies (4.2%) [20]. This further indicates that communities that live in the forested areas, like the ones we studied in western Uganda are at higher risk of infection with filoviruses compared to those living in more developed or non-forested areas. Forested areas tend to have a greater abundance of fruiting trees that provide food to the fruit bats, the hypothesized reservoirs of ebolaviruses. However, in this study, going into the forest was not shown to be a risk factor for individuals being seropositive for filoviruses. While our investigation found that being a miner, but not necessarily living near the mines, is highly associated with being seropositive for filoviruses, it is likely that since the Kitaka caves are in a forested ecosystem near a national park that is comparable to the central African forest, this makes the mines a more attractive location for bats to live. Gold mining has been previously described as a risk factor for Marburg virus infection in a study in DRC [21] with OR = 13.9, 95% CI;3.1–62.1 but not for Ebola virus. We report artisanal mining and going inside the mines as risk factors for being seropositive for filoviruses, including Sudan virus, in Uganda (AOR = 3.4; 95% CI 1.3–8.5). The very first cases of Ebola virus were reported in mining communities in DRC in 1976.

The four seropositive individuals we found in the “unexposed” Luweero district group could be due to travel and migration from high-risk areas but may also be due to the movement of reservoirs such as bats that are known to travel long distances hence spreading the infection. Ebola virus seropositivity has before been reported in a grassland savanna-like ecosystem in Nigeria similar to the grassland savannah ecosystem of Luweero where the three Sudan virus (3) seropositive individuals were identified [22]. However, frequent travels outside high-risk areas were protective (AOR = 0.3; 95% CI 0.1–0.7). This may be because those who frequently travel away from risk areas are less likely to be exposed to the putative reservoir.

Being male was associated with a high risk of being seropositive (AOR = 3.1; 95% CI 1.01–9.5) compared to being female, likely partly due to men being more likely to be miners and go inside the mines and the forests for manual work and become exposed and hence acquire infection. Cleaning a dead body was significantly associated with being seropositive for filoviruses. This has been widely reported in outbreaks of filoviruses as burials and funeral rites amplify these outbreaks. Contact with EVD/MVD suspect was a predictor of filovirus seropositivity and has been reported in a partial meta-analysis done on the risk of ebolaviruses transmissions [23].

Looking at the overall seroprevalence reported in this study, the findings suggest that there may be filovirus infections and outbreaks that occur in Sub- Saharan African countries and go undetected by the health care systems. This could possibly lead to large epidemics as was seen in West Africa [24]. Additionally, our findings do not rule out the possibility that there could be cross-reactivity for filoviruses in our diagnostic assays caused by either another filovirus infection or a non-filovirus infection.

A similar unpublished study was carried out by the CDC and the Ministry of Health following the 2007 MVD outbreak in Kamwenge and Ibanda district in the same area. In that study, they found a seroprevalence of Marburg virus at 1.2% (7/564) and Sudan virus at 1.2% (7/564). The seroprevalence of Marburg virus was slightly lower in our study whereas that of ebolaviruses was higher. We do not have a clear explanation for these differences in seropositivity between the two studies conducted in the same area 8 years apart. Also, the seroprevalence in our study is lower than 8% and 3% for pooled seroprevalences reported in meta-analyses of seroprevalence of ebolaviruses performed in other parts of the world [1, 25]. Following the West Africa EVD outbreak, reports of asymptomatic infection in West African populations has been suggested in populations who had contact with EVD patients at 12%, and at 2.6% in non-contacts [26].

Our investigation also reported a lower seroprevalence of ebolaviruses (2.5%) than pooled seroprevalences reported in other studies in neighbouring Democratic Republic of Congo (DRC) at 10% [19, 27–30], Central African Republic and Gabon at 11% [31–35], Sudan at 22% [36], Madagascar at 4% [11], Liberia at 13% [37] and Cameroon at 7% [38, 39]. However, our study showed higher seroprevalence than that reported in Nigeria at 2% [22], Germany at 1% [12] and Kenya at 1% [13]. Only one Marburg virus seropositive person was confirmed in our study and this was much lower than has been reported in other studies [11–17]. However, further comparison of these studies is difficult due to differences in serological methods used and differences in filovirus species targeted.

These variations in seroprevalence could be due to differences in filovirus ELISA testing protocols and potential cross-reactivity caused by a non-filoviral infection. The test, developed by CDC that was used in this study is more specific than other filovirus serological tests used in previous filovirus seroprevalence studies [9]. This serological test was developed and validated by US Centres for Disease Control and Prevention (CDC) on known positive and negative human samples with a sensitivity > 90% and specificity of > 90%. However, we still see serological cross-reactivity within filovirus species even with this test as was reported by McNeil et al 2011 [40]. For example, one person in this investigation was seropositive for both Sudan virus and Bundibugyo virus, likely a cross-reactivity rather than representing previous exposure to Bundibugyo virus species, as has been described previously [40, 41]. Testing for filoviruses using serological tests can potentially overestimate the true level of seropositivity and therefore overestimate risk and exposure due to varying cross-reactivity between differing and unvalidated serological assays used in previous studies. We are continuing to classify these serological results with validation assays including viral neutralization to confirm if our findings represent true undetected filovirus infections in these communities, or through cross-reaction with other viral infections, or variability in serologic assays performed.

## Conclusions

We conclude that filovirus infections may go undetected by the health care system in Uganda. Also, miners in Ibanda and Kamwenge, near Kitaka mine, are at higher risk of filovirus infection compared to populations living further away from these cave environments. Increased surveillance is still critical in detecting and quickly averting future widespread and devastating filovirus epidemics.

## Data Availability

The datasets used and/or analysed during the current study are available from the corresponding author on reasonable request.

## Abbreviations

EVD: Ebola Virus Disease
MVD: Marburg Virus Disease
ELISA: Enzyme-linked
IgG: Immunoglobulin G
AOR: Adjusted Odds Ratio
VHF: Viral Haemorrhagic Fever
EPI: Expanded Program for Immunization
UVRI: Uganda Virus Research Institute
CDC: United States Centres for Disease Control and Prevention
OR: Odds Ratio
PBS: Phosphate buffered Saline
Ag: Antigen
ABTS: 2,2′-azino-bis (3-ethylbenzothiazoline-6-sulfonic acid)
OD: Optical Density
NTF: National Task Force
SD: Standard Deviation
RR: Risk Ratio
DRC: Democratic Republic Congo
